# Surgical treatment of acute Rockwood III acromioclavicular dislocations—Comparative study between two flip-button techniques

**DOI:** 10.1038/s41598-020-61488-z

**Published:** 2020-03-10

**Authors:** Yu-chen WANG, Yong M. A., Wei-zhong Y. U., Hui WANG

**Affiliations:** 1 Wujin TCM Hospital affiliated to Nanjing University of Chinese Medicine, 699#, Renmin Road, Changzhou, 213161 Jiangsu China; 2 Nanjing University of Chinese Medicine, 210046 Nanjing, China

**Keywords:** Trauma, Outcomes research

## Abstract

Acromioclavicular joint dislocation is a common shoulder injury, usually caused by direct violence on the shoulder. Optimal treatment of type III is still a hot discussion currently in orthopedic surgeons. With the advent of many flip-button techniques, Tightrope system and Endobutton system become popular techniques for reconstruction of coracoclavicular ligaments. The purpose of the study was to compare the clinical and radiological results between the two techniques. A retrospective case-control study was conducted in 60 patients with acute Rockwood III acromioclavicular joint dislocation. The two techniques conducted were open procedures using Twin Tail Tightrope system (Group A, n = 30) and Endobutton system (Group B, n = 30). 60 patients were followed up at least two years. Surgical parameters including incision length, operation time and operative blood loss were analyzed. Functional outcomes were evaluated using the Constant-Murley Score. Radiological results were assessed based on coracoclavicular distance preoperatively, one day postoperatively, and at the final follow-up. 60 patients were followed up for at least 24 months (range 24 to 32). The incision length and operation time were shorter in Group A than that in Group B. The blood loss of surgery was significantly less in the Group A. There were no significant differences between the two groups regarding the Constant-Murley Score at the final follow-up. No significant differences were found in the coracoclavicular distance preoperatively, immediately postoperatively, and at the final follow-up. Both techniques offered satisfying functional outcomes, however the Tightrope system provided better surgical parameters.

## Introduction

Acromioclavicular joint (ACJ) injuries usually occur in a young active population (aged < 35 years) and account for 12% of shoulder injuries^1^. The common nature of injury is a fall or direct trauma to the shoulder. Rockwood classified the dislocations into six types depending on several criteria^2^. Type I is a contusion of the AC ligaments. Type II is a breakage of the AC ligaments and contusion of the coracoclavicular (CC) ligaments, with a slight lateral subluxation of the clavicle and minor enlargement of the CC distance (less than 25%) compared with the unaffected side. Type III is a rupture of both AC and CC ligaments accompanied by 25% to 100% CC distance increase. Type IV is a posterior dislocation of the lateral end of the clavicle into the trapezoid muscle. Type V is a superior dislocation of the lateral end of the clavicle accompanied by 100% to 300% CC distance increase. Type VI is an inferior dislocation below the plane of the coracoid process. Conservative treatments are recommended for Rockwood type I and II, which have been described as low-energy trauma^3^. High-energy trauma as type IV to VI are treated operatively^4^. While the management of type III still remains controversial, which is tending to surgery in younger or functional high demand patients^5^. Many surgical methods have been developed over decades and there still exists a problem concerning the optimal operative treatment between internal fixation or reconstruction of CC ligaments, both of which have their pros and cons^6^. For CC ligaments reconstruction, non-absorbable suture, allogenous/autogenous tendons and the flip-button techniques such as Tightrope system (Arthrex Inc, Naples, FL, USA) and Endobutton system (Smith&Nephew Inc, Memphis, TN, USA) have been widely used^7–11^.

The purpose of the present study is to compare the clinical and radiological results of these two techniques for the treatment of acute Rockwood type III ACJ dislocations.

## Materials and methods

Treatment types are randomly assigned by the computer at admission and the results of the assignment are given to the patient in an opaque envelope. This retrospective study was approved by the Ethical Committee of the Wujin affiliated Hospital of Nanjing University of Chinese Medicine. The study was performed in accordance with relevant guidelines and regulations. Written informed consent was obtained from all patients before surgeries. A retrospective case-control study was performed in patients with acute Rockwood III acromioclavicular dislocation between April 2013 and May 2016. The inclusion criteria were as follows: (1) the acute injury of the ACJ. (2) acute ACJ that occurred less than 3 weeks. (3) radiological results showed Rockwood type III. Exclusion criteria were as follows: (1) previous pathology on the affected shoulder. (2) chronic ACJ dislocations or other types of acute ACJ dislocations. 60 patients were included in the study and allocated to group A and group B.

Group A (Twin Tail Tightrope system) consisted of 30 patients, 18 males and 12 females, aged from 19 to 64 years, with an average age of (39.37 ± 15.31) years. Group B(Endobutton system) consisted of 30 patients, 12 males and 18 females, aged from 20 to 63 years, with an average age of (42.20 ± 13.49) years. The baseline characteristics of each group have no significant difference (Table 1).

**Table 1:** Baseline characteristics of study groups.

Age(years)	Group A(n = 30)	Group B(n = 30)	p
	39.37 ± 15.31	42.20 ± 13.49	0.450
Gender			
male	18	12	0.121
female	12	18	
Injury side			
Left	16	11	0.194
Right	14	19	
Cause of injury			
Traffic	8	12	0.379
Sports	9	5	
Electric scooter	13	13	
Time from injury to surgery(days)	5.30 ± 1.42	5.17 ± 1.51	0.726
Follow-up(months)	27.67 ± 2.48	28.30 ± 2.51	0.330

Patients were followed up for at least two years. Constant-Murley Score (CMS) was applied to evaluate pain, activities, range of movement, powers of muscle postoperatively. Radiological results were assessed based on coracoclavicular distance preoperatively, one day postoperatively, and at the final follow-up.

### Surgical methods

All patients were positioned in the beach chair under general or brachial plexus anesthesia.

**Figure 1:**
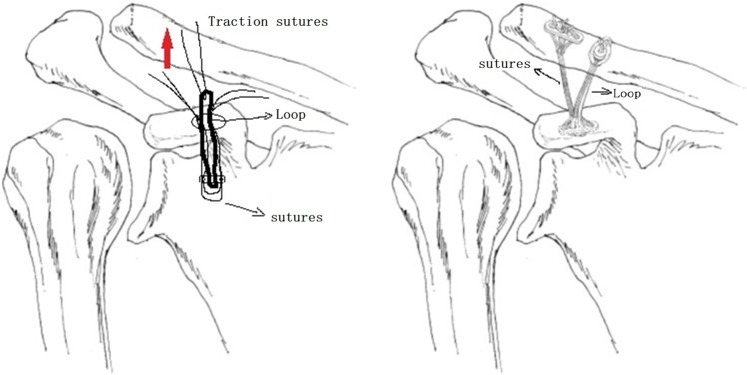
Schematic drawing of Endobutton.

Group A: A transverse incision was made from the ACJ to proximal anterior clavicle, the deltoid was dissected off to view the base of the coracoid process. A 3.0 mm drill was used to center on top of the base of coracoid to establish the coracoid tunnel. The coracoid button was pushed through the coracoid tunnel by button inserter. All sutures were grasped and pulled proximally to flip the coracoid button. a 2 mm K-wire was used to drill clavicle tunnels, which located at the midline approximately 45 mm and 20 mm from the end of the clavicle. The reduction of ACJ was accomplished by pulling on the sutures sequentially. Once the position of the implant and reduction of AC joint are confirmed to be satisfactory on fluoroscopy, the sutures were tied (Fig. 1).

**Figure 2:**
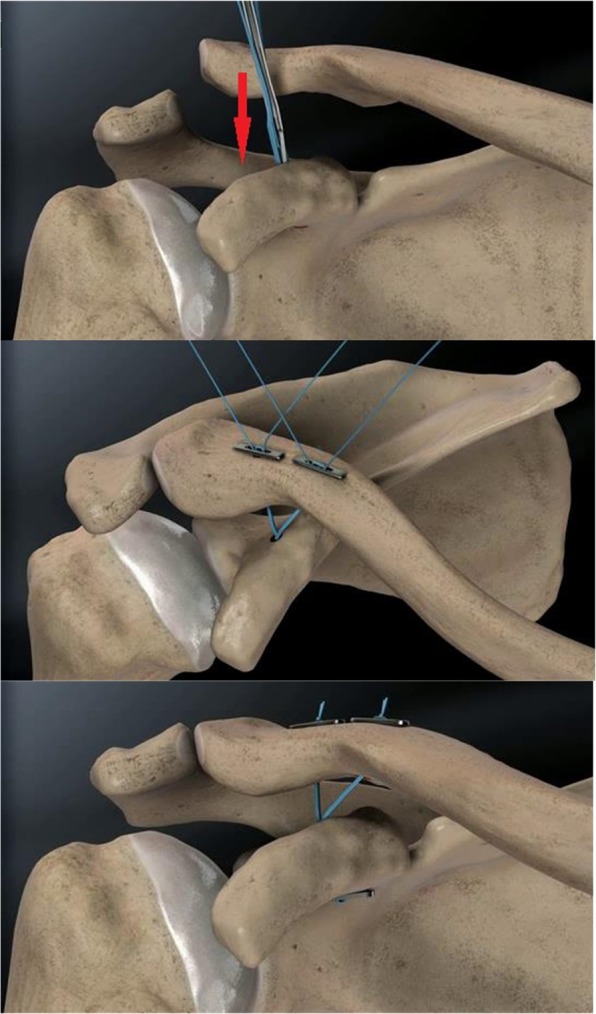
Schematic drawing of Twin Tail TightRope.

Group B: A straight incision was made spanning the distal clavicle to proximal to the coracoid. The deltoid was dissected off the anterior-superior clavicle subperiosteally, which allowed exposing the base of coracoid and the distal clavicle. A 4.5 mm drill was well-centered between the medial and lateral edges of coracoid to establish the coracoid tunnel. The clavicular tunnels were created by 2.0 mm K-wire at superior surface of the clavicle approximately 45 mm and 20 mm medial to the end of the clavicle. Two strands of Ethibond (Johnson & Johnson, New Brunswick, NJ, USA) sutures were placed through the first and fourth holes of the coracoid button to reconstruct trapezoid portion of CC ligament. One strand of FibreWire suture (Smith&Nephew Inc, Memphis, TN, USA) was placed through the closed loop as traction suture. All sutures and loop were pulled through the coracoid tunnel from the bottom and the coracoid button was flipped and set underneath the coracoid process. When the loop just came out from the top of the clavicle tunnel, a clavicle button was inserted into it and tied. Two strands of Ethibond suture are passed through the lateral clavicle tunnel. Then the suture tails were passed through the holes of the second clavicle button and tied (Fig. 2).

### Rehabilitation and follow-up

All patients were managed by simple sling immobilization for at least two weeks. Exercise of upper limb joints was required in the first four weeks of rehabilitation. It was recommended to begin passive motion of the shoulder four weeks after surgery. Physical examination was conducted and the X-rays were taken to measure the CC distance. The function of the shoulder was evaluated by using the CMS.

### Radiographic assessment

Radiological follow-up was at 4, 8 and 12 weeks and then at 12 months and finally at the last follow-up. Anteroposterior and stress radiographs views of both shoulders were obtained. Diagnosis of acute AC dislocation Rockwood type III was based on the clinical and radiographic examination of calculated AC and coracoclavicular distances compared with the contralateral side. The postoperative CCD increased by 50% over healthy side was defined as radiological failure.

### Statistical analysis

SPSS 19.0 software (SPSS Inc, Chicago, IL, USA) was used for all statistical analyses. Normally distributed data were presented as mean ± SD using the Kolmogorov-Smirnov test. Statistical testing utilized unpaired t-test for continuous variables, χ^2^ test and Fisher’s exact test for categorical variables. Statistical significance was defined as p < 0.05 (two-tailed).

## Results

### Clinical and radiographic outcomes

Patients visiting the hospital from April 2013 to May 2016 with acute Rockwood III acromioclavicular dislocations were considered for inclusion in the study. 60 patients were followed up for 27.9 months (range, 24 to 32). X-rays of all affected shoulders presented reduction of ACJ one day after surgery.

Incision Length in Group A was (4.34 ± 0.82) cm (range, 3.2–5.8), which was shorter than that in Group B (6.43 ± 0.86) cm (range, 5.3–8.5). The Operational time was shorter in Group A, in which the time was (32.20 ± 5.47) min (range, 25–41), compared with Group B, in which the time was (47.60 ± 3.95) min (range 40 to 55). The blood loss was less in Group A with an average amounts of (44.97 ± 9.47) ml (range 30 to 60) compared with an average amounts of (74.70 ± 15.32) ml (range 50 to 100) (Table 2).

**Table 2:** Comparison of surgical parameters.

	Group A(n = 30)	Group B(n = 30)	p
Incision length (cm)	4.34 ± 0.82	6.74 ± 0.83	<0.01
Operational time(min)	32.20 ± 5.47	47.60 ± 3.95	<0.01
Blood loss(ml)	44.97 ± 9.47	74.70 ± 15.32	<0.01

There was no statistically significant difference in preoperative CMS of shoulder and CC distance between the two techniques. In Group A the CMS improved from 69.57 ± 3.10 (range, 65–75) preoperatively to 93.70 ± 1.78 (range, 91–96) at the final follow-up (p < 0.001). In Group B the CMS improved from 69.73 ± 3.29 (range, 65–75) preoperatively to 93.27 ± 1.59 (range, 91–96) at the final follow-up (p < 0.001). There was no statistically significant difference regarding the CMS at the final follow-up between the two groups(p = 0.326).

At the one day postoperative and final follow-up, no difference was found between the two groups regarding the CC distance (p > 0.05; Table 3). Three cases(1 in Group A, 2 in Grope B) suffered slight loss(increased < 50% normal CC distance).

**Table 3:** Comparison of functional and radiological results.

	Group A(n = 30)	Group B(n = 30)	p
Constant-Murley Score			
Preoperative	69.57 ± 3.10	69.73 ± 3.29	0.841
Final follow-up	93.70 ± 1.78	93.27 ± 1.59	0.326
Coracoclavicular distance			
Preoperative	23.50 ± 2.08	23.57 ± 2.69	0.915
1 day post operation	10.57 ± 1.14	10.47 ± 1.15	0.734
Final follow-up	11.40 ± 1.13	11.47 ± 1.19	0.825

### Complications

On the 2 years follow-up, no complications including wound infection and neural injury were found in both groups. Postoperative complications occurred in only one patient in Group B. the patient suffered a breakage of loop 3 months postoperatively, which led to re-dislocation of ACJ. The patient accepted a revision surgery using the Tightrope system.

### Subgroup analysis

A few cases (n = 17) had a horizontal displacement, so we corrected it with horizontal cerclage. Subgroup analysis of this group of patients found that there was no statistical difference in terms of CMS and CCD among subgroups (Table 4).

**Table 4:** Subgroup analysis of horizontal cerclage.

	horizontal cerclage	Non- horizontal cerclage	P
	n = 17	n = 33	
CMS			
Preoperative	66.32 ± 3.10	68.54 ± 2.58	0.735
Final follow-up	92.17 ± 1.11	93.12 ± 1.54	0.456
CCD			
Preoperative	22.23 ± 1.98	23.11 ± 1.56	0.875
1 day post operation	9.87 ± 1.04	10.01 ± 1.05	0.694
Final follow-up	11.38 ± 1.03	12.01 ± 1.35	0.795

## Discussion

Two portions of the CC ligament maintain the stability of ACJ. Debski *et al*.^12^ proved the conoid ligament restrains the movement against anterosuperior force of clavicle and the trapezoid ligament against the posterior force of clavicle. The Weaver-Dunn and Bosworth techniques, with their various modifications, are currently the most popular procedures in use^13,14^, alongside various fixation methods, like screws, pins, hook plates. Recently, hook plate is widely used. The hook end of the plate underneath the acromion may lead to shoulder pain, subacromial impingement and damage on the supraspinatus tendon. Plate retention may cause acromial osteolysis or fracture^15^. It is necessary to remove the plate after 3 to 6 months when the ligaments are restored. Kienast *et al*.^16^ performed good outcomes of reduction using the hook plate in 313 patients of Rockwood type III–V ACJ dislocation, while almost all patients reported pain or discomfort after surgery. A high incidence of complications of soft tissue infections, fracture of the acromion and loss of reduction were reported after removal of the hook plate^17^.

Internal fixation can only achieve a reduction of ACJ temporarily, which will increase the risk of re-dislocation. The stability of anatomical structures of ACJ still relies on those surrounding ligamentous tissues, especially the CC ligaments. Therefore, reconstructing CC ligament as close to the original anatomy as possible may stabilize the ACJ and improve the shoulder function. A biomechanical study performed by Saier *et al*.^18^ suggested that combined AC and CC reconstruction can adequately re-establish physiological horizontal ACJ stability. Therefore, it is likely that a combined surgical procedure with double suture-button devices and AC suture tape cerclage can adequately re-establish horizontal AC joint stability.

Ligamentous reconstruction method as the flip-button technique precedes conventional internal fixation in some aspects^4^: it will regain biomechanical stability of ACJ, and avoid interference of soft tissues and ligaments around acromion. It allows early functional exercise for patients and accelerates the recovery of shoulder function. It permits micro-motion of ACJ which conforms to biomechanical principle. The technique renders it unnecessary to remove the implants, which is cost-effective.

The Endobutton system is a device initially designed for anterior cruciate ligaments graft fixation^19^. It consists of one-size titanium button and multiple, pre-measured loop sizes. The triple Endobutton technique applying for coracoclavicular (CC) ligament reconstruction was first reported by Lim^20^. The Twin Tail Tightrope system features two independent clavicle button tails and is designed to help reduce and stabilize the ACJ. Each clavicle button is independently joined the coracoid button with a continuous loop of FiberWire. The twin tails enable the surgeon to stabilize the ACJ with a device that matches the normal CC ligament anatomy.

The study compared the surgical outcomes between Twin Tail Tightrope system and Endobutton system for treatment of acute Rockwood type III AC dislocations. Both techniques can provide satisfactory postoperative shoulder function and low incidence rate of reduction loss. There is an imperfection that the length of closed loop is prefabricated, in some cases of our own, inaccurate selections not only caused more operation time and blood loss, also led to loss of reduction or over-reduction of ACJ.

As to the incision length, how the flip-button is set underneath the coracoid process determines the incision location and exposure area. The coracoid button of Endobutton system is pulled from bottom of coracoid while that of Twin Tail Tightrope system is inserted from above to below of coracoids. In view of this, we just need to make a short transverse incision on anterior clavicle, and a slight dissection of the tissues above coracoid base, which is less invasive.

Using Twin Tail Tightrope system, we achieved anatomical reduction right after the operation in all cases without differences compared with Endobutton system. In our series we discovered three cases (10%) with a slight loss of reduction in the study during the first three months, one in Group A (7.1%) and two in Group B (12.5%). No further loss of reduction was observed after that which proved the significance of proper restraint in the early stages of treatment. The results in Group A showed significant differences compared with literature^21^. This difference shows that the stability of twin tails device is better than a single tail.

According to the CMS, there was no significant difference between the two methods. In the meantime, their CMS showed similar outcomes compared with other literatures^22^.

## Conclusion

The two techniques offered satisfying clinical outcomes, but Twin Tail Tightrope system was associated with a shorter operative procedure, a smaller scar and less blood loss.
